# Metagenome-assembled genome of *Synechococcus* sp. “Tanasi” from the Tellico Reservoir, Tennessee, USA

**DOI:** 10.1128/mra.00417-25

**Published:** 2025-07-23

**Authors:** David J. Niknejad, Robbie M. Martin, Emily E. Chase, Randall H. Morse, Gary M. Lucas, Jennifer M. DeBruyn, Forbes R. Walker, Eric A. Webb, Steven W. Wilhelm

**Affiliations:** 1 Department of Microbiology, University of Tennessee 189504 , Knoxville, Tennessee, USA; 2 Watershed Association of the Tellico Reservoir, Lenoir City, Tennessee, USA; 3 Department of Biosystems Engineering and Soil Science, University of Tennessee 4292, Knoxville, Tennessee, USA; 4 Department of Biological Sciences, University of Southern California 118558, Los Angeles, California, USA; California State University San Marcos, San Marcos, California, USA

**Keywords:** cyanobacteria, freshwater, metagenomics

## Abstract

*Synechococcus*-like cyanobacteria are vital to aquatic ecosystems. Taxonomists have begun to reassign genomic clades within this group to new taxa. This process depends on genomic data from diverse habitats. Here, we present a metagenome-assembled genome of a *Synechococcus* sp. strain from the Tennessee River Basin, an ecosystem under-represented in databases.

## ANNOUNCEMENT

Picocyanobacteria, especially members of the *Synechococcus* collective (1), contribute to primary productivity, food webs, and biogeochemical cycles of fresh and marine waters worldwide (2). The *Synechococcus* collective is globally distributed and abundant in lakes and reservoirs (2). The taxonomic assignment of *Synechococcus* is convoluted, and organisms previously assigned to this genus were polyphyletic (1). Taxonomists are reassigning genomic clades within this group to new monophyletic genera, but this process depends on the acquisition of genomic information from diverse environments inhabited by *Synechococcus*-like organisms (3).

The Tennessee River Basin is a hotspot of aquatic biodiversity (4–6), yet the microbial diversity of this ecosystem is virtually unexplored. Tellico Reservoir is a 15,560-acre impoundment within the Tennessee River Basin formed in 1979 by damming the Little Tennessee River. We present a metagenome-assembled genome (MAG) of *Synechococcus* sp. Tanasi collected from the Tellico Reservoir, Tennessee, USA. The strain was named after a Cherokee Nation settlement now submerged beneath the Tellico Reservoir (7).

Samples for metagenomic sequencing were collected at 0.5 m on 21 July 2022 and 31 August 2022, near Little Tennessee River mile 12.0 (35°39.558N, 84°15.120W) and on 21 July 2022, near river mile 11.5 (35°39.518N, 84°15.969W). Approximately 240 mL of water was filtered through 0.22 µm Sterivex filters. Filters were immediately frozen in liquid nitrogen. DNA was extracted from filters using the phenol-chloroform method (8). Using 400–450 ng total DNA, libraries were prepared at the SeqCenter (Pittsburgh, PA) using the Illumina DNA Prep Kit with IDT 10 bp UDI indices and sequenced on the Illumina NextSeq 2000 platform, producing 2 × 151-bp-PE reads.

Default parameters were used in bioinformatic software unless specified. A total of 25,595,806 reads from the three libraries were pooled and assembled with metaWRAP (v1.3.2) (9) using Megahit (v1.1.3) (10). Assembled contigs were binned using the metaWRAP package with metaBAT2 (v2.12.2) (11), MaxBin2 (v2.2.6) (12), and CONCOCT (v1.0.0) (13). Bin quality was assessed using CheckM2 (v1.0.1) (14), BUSCO (v5.7.1) (15), and QUAST (v5.2.0) (16). Genome completeness was estimated with CheckM2. Taxonomy was assigned using GTDB-Tk (v2.3.2) employing the R07-RS207 database release (17). Average nucleotide identity (ANI), calculated by FastANI (v0.1.3) (18), provided insight into the relatedness of our MAG to other genomes. Genomes for ANI comparison were selected from hits in GTDB, those used in the “Insert Genome Into Species Tree” (v2.2.0) in KBase (19), and by manual searches in GenBank. The genome was annotated with PGAP (v6.9) (20). A phylogenetic tree of related genomes was built using GToTree (v1.8.6) (21) and IQ-TREE (v2.3.6) (22) and visualized using iTOL (v6) (23).

**Fig 1:**
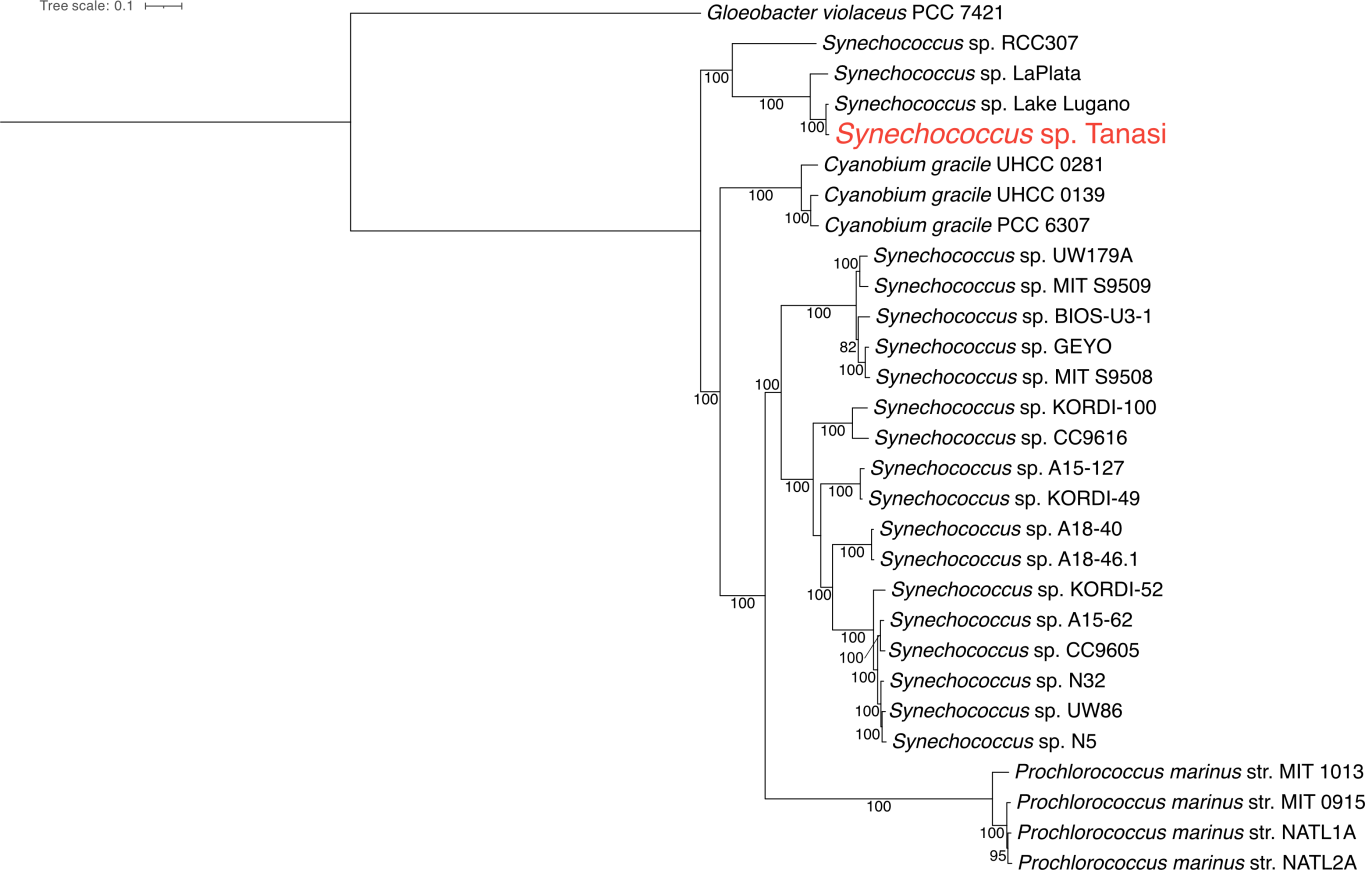
Maximum likelihood phylogenetic tree of closely related cyanobacteria and the MAG presented in this paper, constructed by IQ-TREE (v2.3.6). The tree was rooted by *Gloeobacter violaceus* PCC7421. A best-fitting model was selected for each marker gene using ModelFinder Plus, and 1,000 ultrafast bootstraps were used to construct this tree. Bootstrap values are indicated at the nodes. Taxa included in the tree were selected from Lee et al. (26), with *Prochlorococcus* spp. sequences added to confirm this distinct lineage, plus the Lake Lugano and LaPlata MAGs, which were identified by GTDB-Tk and ANI as most closely related to our MAG.

The *Synechococcus* sp. Tanasi MAG consists of 44 contigs (N_50_ = 92,698 bp) with a total length of 2,535,063 bp and a GC content of 67.64%. It is estimated to be 98.5% complete with 0.05% contamination and contains 2,700 putative protein-encoding and 45 RNA-encoding genes. The most closely related genomes are a *Synechococcus* sp. MAG from Lake Lugano, Italy (ANI = 96.8%) (24) and an “uncultured cyanobacterium” from LaPlata, Puerto Rico (ANI = 86.8%) (25). These three genomes clustered together (Fig. 1).

## Data Availability

The genome is deposited at DDBJ/ENA/GenBank under accession JBMDLR01. Reads are deposited in the NCBI SRA under BioProject PRJNA1202192 and SRX27173386, SRX27173385, and SRX27173384.
